# Beads-Based Electrochemical Assay for the Detection of Influenza Hemagglutinin Labeled with CdTe Quantum Dots

**DOI:** 10.3390/molecules181215573

**Published:** 2013-12-13

**Authors:** Ludmila Krejcova, Lukas Nejdl, David Hynek, Sona Krizkova, Pavel Kopel, Vojtech Adam, Rene Kizek

**Affiliations:** 1 Department of Chemistry and Biochemistry, Faculty of Agronomy, Mendel University in Brno, Zemedelska 1, Brno CZ-613 00, Czech Republic; 2 Central European Institute of Technology, Brno University of Technology, Technicka 3058/10, Brno CZ-616 00, Czech Republic

**Keywords:** influenza hemagglutinin, electrochemical detection, paramagnetic particles, glycan, quantum dot labeling

## Abstract

In this study we describe a beads-based assay for rapid, sensitive and specific isolation and detection of influenza vaccine hemagglutinin (HA). Amplification of the hemagglutinin signal resulted from binding of an electrochemical label as quantum dots (QDs). For detection of the metal and protein part of the resulting HA-CdTe complex, two differential pulse voltammetric methods were used. The procedure includes automated robotic isolation and electrochemical analysis of the isolated product. The isolation procedure was based on the binding of paramagnetic particles (MPs) with glycan (Gly), where glycan was used as the specific receptor for linkage of the QD-labeled hemagglutinin.

## 1. Introduction

These days are characterized by rapid population growth, international trade and globalization, and for this reason the risk of influenza pandemics looms larger and larger [1]. Influenza is one of the most frequently occurring respiratory diseases, which causes approximately 500,000 deaths every year. The influenza virus is subject to genetic mutations, mainly due to the lack of proof-reading activity of its polymerase [2]. Influenza infections mostly occur as seasonal epidemics or less frequently, as influenza pandemics. During twentieth century there were three, with shortening delays between them: Spanish flu in 1918, Asian flu in 1957 and Hong-Kong flu in 1968. The twenty first century has also been marked by a few cases of pandemics (swine and avian influenza) with a small number of victims, but with much larger economic impact.

Antigenic drift results from an accumulation of point mutations leading to minor antigenic changes, while antigenic shift involves major antigenic changes by introduction of new hemagglutinin (HA) and/or neuraminidase (NA) subtypes into the human population [2]. Combinations of HA (1-17) and NA (1-9) subtypes affect the biological properties of influenza viruses, especially their host range and virulence. HA is responsible for the initiation of the infection and it binds sialic acid on the host cell surface. NA removes sialic acid from the host receptor and thus enables the release of the replicated virion [3,4]. Sialic acids (SAs) are located on the terminal positions of glycans on the host cell surface, which is important for the spread of pathogens [5,6].

Vaccination is the most effective way of preventing influenza epidemics and the emergence of pandemics [7,8,9], however, influenza vaccines are only effective one year, because the limiting factors are the antigens’ mutational changes, which mean that reuse of the previous year’s vaccine would not be effective [10]. In 2006, World Health Organization (WHO) published an action plan to increase the current supplies of influenza vaccines. Inactivated vaccines induce protective levels of serum antibodies to influenza HA surface proteins [9] and are regarded as the most effective prophylactic method. Before the presentation of the influenza vaccine on the pharmaceutical market rapid and sensitive analysis of vaccination antigens is necessary, especially for human immunization. As a detection system, electrochemical biosensors and bioassays have attracted considerable interest due to their high performance, miniaturized construction, and low costs [11,12,13,14,15]. In spite of the fact that quantum dots (QDs) and magnetic nanoparticles (MPs) are currently utilized, especially for *in vivo* and *in vitro* imaging, their application in the field of biosensing devices for influenza viruses could be also considered [16,17,18]. 

The aim of this study was to design and to test a method for indirect detection of hemagglutinin for the purposes of quantification of vaccine antigen or for the detection of influenza virus. We suggest a method based on the isolation of QD-labeled HA by glycan-modified MPs and on electrochemical detection of the MPs-HA-glycan-QDs complex, which is formed due to the specific and selective binding between glycans and HA.

## 2. Results and Discussion

Influenza type A can be subtyped by different variants of NA (1-9) and HA (1-17), which differ from each other in virulence, host specificity and binding of HAs subtypes on human and/or avian glycan receptors [19,20,21,22,23,24]. Specific HA-glycan affinity has been exploited as the basis for isolation of various types of influenza viruses [25]. In our previous study we described a MPs-based isolation method for standard viral proteins labeled with CdS [26]. The current study was moved forward by the isolation of real samples (influenza vaccines) instead of the HA standard. Labeling of HA with CdTe QDs was used because of their better electrochemical properties.

### 2.1. Characterization of vaxi HA, CdTe and HA-CdTe Complex by Gel Electrophoresis

**Figure 1:**
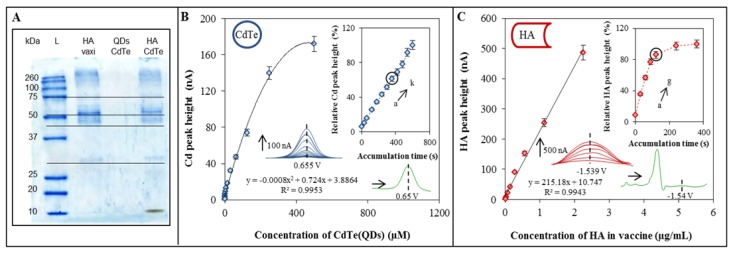
Characterization of QDs and hemagglutinin by gel electrophoresis and electrochemical analysis. (**A**) Characterization of vaxi HA, CdTe and vaxi HA-CdTe by gel electrophoresis (SDS PAGE). Concentration of labeled and non-labeled vaxi HA was 22.5 µg/mL, concentration of CdTe was 500 µM; (**B**) Electrochemical detection of individual parts of HA-CdTe complex. Dependence of Cd peak height on Cd (CdTe) concentration. Inset: dependence of relative Cd peak height (related to the maximum value) on time of accumulation. Cd was determined by DPASV with parameters as it follows: initial potential −0.8 V; end potential -0.5 V; deposition potential -0.8 V; equilibration time 5 s; modulation time 0.06 s; time interval 0.2 s; potential step 0.002 V; modulation amplitude 0.025 V. Time of accumulation was optimized (a → k): *a* 30 s, *b* 60 s, *c* 120 s, *d* 180 s, *e* 240 s, *f* 300 s, *g* 360 s, *h* 420 s, *i* 480 s, *j* 540 s, and *k* 600 s; (**C**) Dependence of HA peak height on HA (vaxi HA) concentration. Inset: dependence of relative HA peak height (related to the maximum value) on time of accumulation. For measurement AdT DPV Brdicka reaction was used under following parameters: purge time 30 s; initial potential −0.7 V; end potential −1.8 V; potential step 0.002 V; amplitude 0.025 V. Time of accumulation was optimized (a → g): *a* 0 s, *b* 30 s, *c* 60 s, *d* 90 s, *e* 120 s, *f* 240 s, and *g* 360 s. Concentration of vaxi HA was 2.25 µg/mL, initial concentration of CdTe was 500 µM.

The beads-based isolation of HA-CdS complex is the cornerstone of the proposed procedure. Glycan-conjugated (modified) beads bind vaccine HAs, which could be recognized specifically and linked onto the surface of the glycan-modified MPs. This design is based on the hemagglutinin as the basic element. Therefore the protein characterization of influenza vaccine by gel electrophoresis is very important. The complexity of work with such sample is due to the many proteins present in samples. The interpretation of the gel electropherogram (Figure 1A) is based on the results presented in literature [27,28,29]. The proportions of several influenza proteins have been found to be similar for various strains of the virus [28]. One band at 28 kDa corresponds to the matrix protein 1 (M1). The band at 38 kDa represents the hemagglutinin fragment from influenza B virus. The band at 48 kDa was assigned to the glycosylated HA [30]. The band at 56 kDa is connected with the presence of nucleocapsid protein (NP). The predicted molecular mass of HA is approximately 63 kDa, however, the band pattern of HA is affected by glycosylation and proteolytic cleavage under *in vivo* conditions [31].

### 2.2. Characterization of CdTe by Electrochemical Analysis

Further, we aimed our attention at determination of the metal part of the label used in this study to mark the virus. Cd in CdTe and HA-CdTe complex was determined by differential pulse anodic stripping voltammetry (DPASV) at the potential −0.66 ± 0.005 V (bottom inset in Figure 1B). Optimization of the electrochemical procedure was done due to the testing of accumulation time (upper inset in Figure 1B). Dependence of relative Cd peak height (%) on time of accumulation is linear within the whole testing interval from 0 to 600 s. As the optimum time, 360 s was selected (due to the time consuming nature of measurements with longer accumulation times). The dependence of the peak height on concentration of CdTe had a sigmoidal shape with the following regression curve: Cd in CdTe y = −0.0008x^2^ + 0.724x + 3.8864, R² = 0.9953, n = 4 (Figure 1B). 

### 2.3. Characterization of Influenza Vaccine by the Brdicka Reaction

Proteins from influenza vaccine were characterized by differential pulse voltammetry (AdT DPV) measured in the Brdicka solution and coupled with the adsorptive transfer technique. As the targer peak the so called Cat2 peak at potential −1.56 ± 0.005 V was choosen (bottom inset in Figure 1C). Apply the same concept as above, we also optimized the time of accumulation. From the obtained dependence of relative detected peak height (%) on time of accumulation, 120 s was selected (upper inset in Figure 1C) as the optimum. The calibration curve of the obtained concentration dependence was as follows: y = 215.18x + 10.747, R² = 0.9943, n = 4 (Figure 1C).

### 2.4. Electrochemical Characterization of HA-CdTe Complex

**Figure 2:**
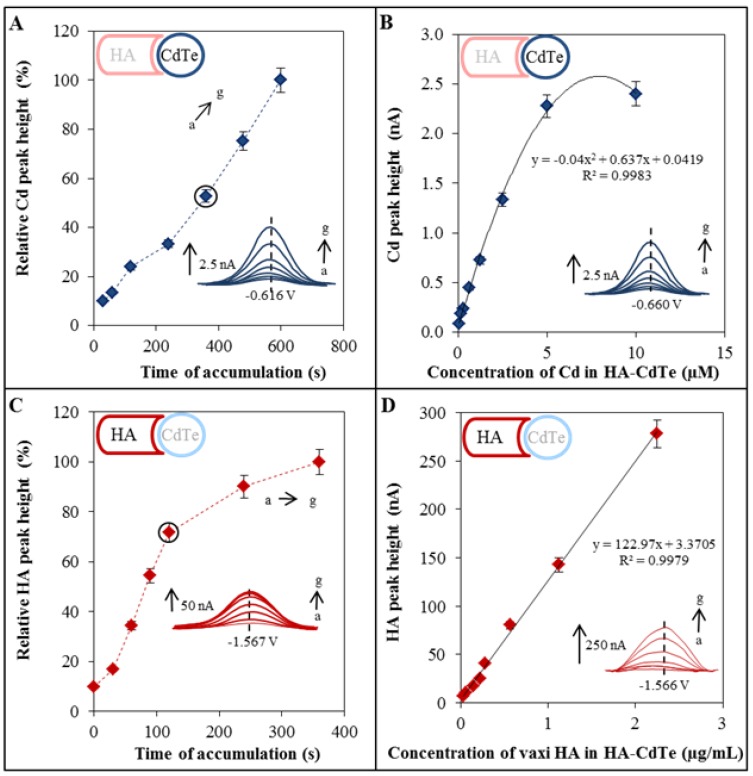
Electrochemical characterization of HA-QDs complex. *Optimization and detectionof the metal part of HA-CdTe complex*. (**A**) Dependence of relative Cd peak height (related to the maximum value) on time of accumulation; (**B**) Dependence of Cd peak height on concentration of CdTe. For Cd determination DPASV was used under the following parameters: initial potential −0.8 V; end potential −0.5 V; deposition potential −0.8 V; equilibration time 5 s; modulation time 0.06 s; time interval 0.2 s; potential step 0.002 V; modulation amplitude 0.025 V. Time of accumulation was optimized (a → g): *a* 30 s, *b* 60 s, *c* 120 s, *d* 240 s, *e* 360 s, *f* 480 s, and *g* 600 s. *Optimization and detection of HA (protein part of the HA-QDs complex)*; (**C**) Dependence of relative HA peak height (related to the maximum value) on time of accumulation; (**D**) Dependence of HA peak height on HA(HA-QDs) concentration. For HA determination AdT DPV Brdicka reaction was used under the following parameters: purge time 30 s, initial potential −0.7 V; end potential −1.8 V; potential step 0.002 V; amplitude 0.025 V. Time of accumulation was optimized (a → g): *a* 0 s, *b* 30 s, *c* 60 s, *d* 90 s, *e* 120 s, *f* 240 s, and *g* 360 s.

HA-CdTe complex formed due to the specific and selective binding between glycan and HA was characterized by two electrochemical methods. Each method serves for the detection of one part of the complex. Characterization of the metal part of the complex, namely the cadmium ions, was done by the DPASV method. The cadmium peak was detected at the potential −0.66 V. For this purpose the method was optimized through determination of the optimal accumulation time (Figure 2A). As it was mentioned above, the value of 360 s was confirmed. Concentration dependence was as follows: y = −0.04x^2^ + 0.637x + 0.0419, R² = 0.9983, n = 4 (Figure 2B).

Characterization of HA part of the complex was done by the AdT DPV method connected with the Brdicka reaction. The detected peak was recorded at a potential near −1.56 V. The method optimization included the optimal accumulation time determination too (Figure 2C). As it was mentioned above, the value of 120 s was confirmed. The calibration curve of the obtained concentration dependence was as follows: y = 122.97x + 3.3705, R² = 0.9979, n = 4 (Figure 2D).

### 2.5. Optimization of the Automated Isolation Procedure

**Figure 3:**
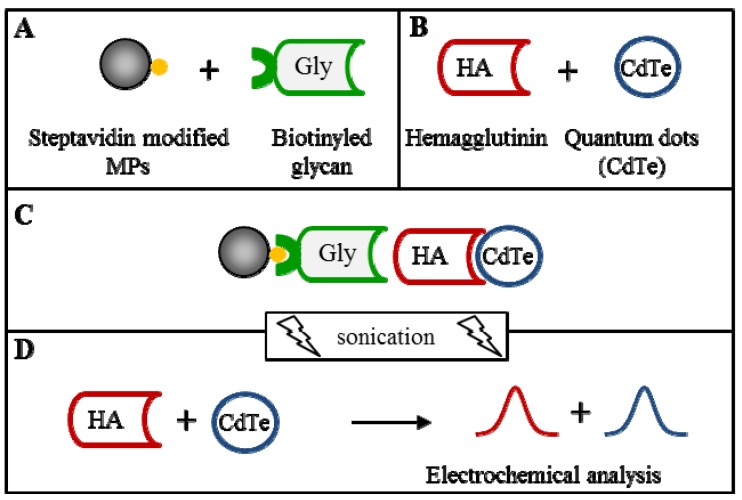
Scheme of isolation (**A**+**B**+**C**) and electrochemical detection (**D**) of vaccine hemagglutinin (vaxi HA) labeled with CdTe quantum dots (QDs); (**A**) Biotinyled glycan binding on streptavidin modified paramagnetic particles (MPs) based on biotin-streptavidin affinity, (**B**) HA labelling by CdTe, (**C**) magnetic isolation of HA-CdTe complex (based on glycan-HA affinity), followed by sonication and (**D**) electrochemical detection of HA and QDs parts. HA was detected by differential pulse voltammetry (DPV) connected with adsorptive transfer technique (AdT DPV) Brdicka reaction. QDs (Cd respectively) were detected by DPASV. Other experimental conditions see in Figure 2.

The isolation procedure was done according to the previously published paper [26]. The isolation procedure scheme is shown in Figure 3. Briefly, streptavidin-modified MPs bind biotinyled glycan and in the other step the hemagglutinin binds quantum dots. In the next step the final complex is created from all four parts. This complex is subsequently disordered by sonication and the individual parts are detected.

**Figure 4:**
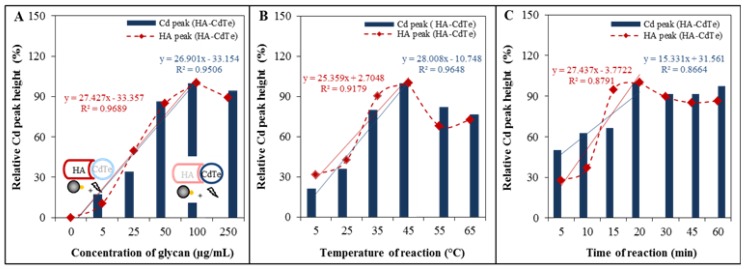
Optimization of isolation procedure. (**A**) Dependence of relative Cd peak height (related to the maximum value) on optimized condition (blue column). Dependence of relative HA peak height (related to the maximum value) on optimized condition (red rhomb). Dependence of relative Cd/HA peak height on concentration of glycan; (**B**) Dependence of relative Cd/HA peak height on temperature of reaction between HA-CdTe and glycan modified MPs; (**C**) Dependence of relative Cd/HA peak height on time of reaction (HA-CdTe and glycan-MPs). Other experimental conditions see in Figure 2.

This procedure was optimized to increase the HA-CdTe yield. The effect of different conditions such as the amount of glycan, time and temperature of isolation was tested using an automatic isolation procedure performed by an ep*Motion* 5075 device. The influence of different conditions was investigated by the optimized electrochemical detection. The effect of glycan amount was investigated first. Figure 4A shows the increasing dependency of HA and Cd relative peak height on the glycan amount, but only up to a concentration of 50 µg/mL (this concentration was established as the best). Blue columns show the dependence of Cd relative peak height on glycan concentration, and red rhombi show the dependence of HA relative peak height on glycan concentration. The other two conditions optimized directly included the labeled HA isolation reaction. Tested conditions included six temperatures (5, 25, 35, 45, 55 and 65 °C) and reaction times (5, 10, 15, 20, 30, 45 and 60 min). From the results it follows that best signals were obtained at temperatures higher than 35 °C and the temperature of 45 °C was established as the best, based on the Cd peak results (blue columns in Figure 4B) and HA peak (red rhombi in Figure 4B), which correspond to the range of body temperatures of humans and small animals, especially birds, which serve as influenza reservoirs [32]. The highest effect of reaction time was observed based on the Cd peak (blue columns in Figure 4C) and HA peak (blue columns in Figure 4C) after 45 min of incubation of glycan-modified MPs with HA-Cd-Te. If we summarize the results of the optimization, the best conditions was glycan concentration 50 µg/mL, reaction time of 45 min., and the reaction temperature 45 °C. The observed distinct temperature dependence of HA-Cd-Te interaction is in accordance with previously published data on the thermostability of hemagglutinin, whereby at temperatures higher than 60 °C the loss of HA secondary structure occurs and at temperatures below 30 °C HA conformational changes affecting the functionality of the virion are observed [28,33,34]. 

### 2.6. Hemagglutinin Detection from the Influenza Vaccine

**Figure 5:**
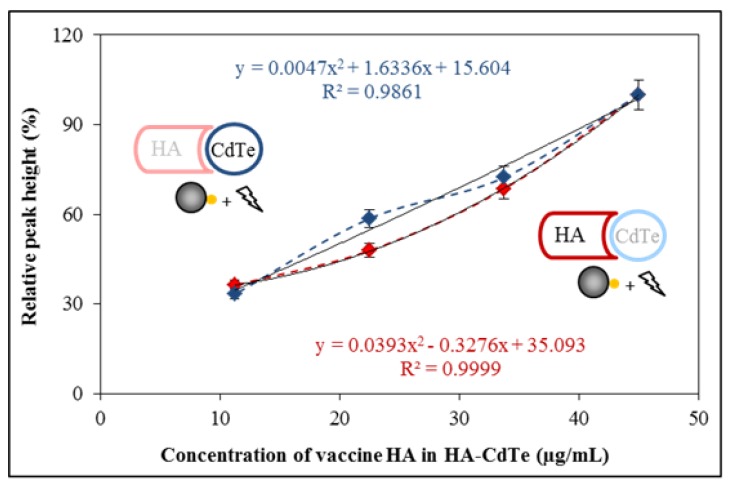
Detection of isolated HA-CdTe complex by electrochemical analysis. Dependence of relative peak height (related to the maximum value, Cd peak - blue colour, HA peak - red colour) on concentration of isolated HA-CdTe complex. Other experimental conditions see in Figure 2.

Usage of influenza vaccine as a source of hemagglutinin for the proof of the suggested concept was tested. The whole isolation procedure was done according to the optimized conditions and as the next step the HA-CdTe complex was detected. Electrochemical detection was applied to both parts of the complex. The dependencies of the individually detected electrochemical signals on the concentration of HA-CdTe complex are shown in Figure 5. For electrochemical determination two different electrochemical methods were utilized, individually focused on the Cd and HA part of the complex. Both dependencies have polynomial character. The CdTe part had dependence as follows: y = 0.005x^2^ + 1.634x + 15.60, R^2^ = 0.986, n = 4. The HA part had a dependence as follows: y = 0.039x^2^ − 0.328x + 35.09, R^2^ = 0.999, n = 4. The lowest concentration of HA in the vaccine (10 µg/mL) refers to 0.2 µmol/L HA. When electrochemical detection of HA-CdTe was tested, the lowest detectable concentration was 62.5 pmol/L. The obtained results are comparable with other nanoparticle-based influenza (immuno)sensors with terminal HA quantification [15], such as an electrically active magnetic nanoparticle-based biosensor for the detection of pandemic influenza with a detection limit of 1.4 µmol/L [35] or the detection of the hemagglutinin molecules of influenza virus by combining an electrochemiluminescence sensor with an immunoliposome encapsulating a Ru complex with a detection limit on the order of atomoles per 50 µL of sample volume, which corresponds to 120 pmol/L [36]. The vaccine is prepared by splitting and inactivating influenza virions. If the vaccine HA is able to immunize humans, the structure of the HA would not be significantly changed, and it will be possible to also apply this method for the isolation and detection of influenza virions. For this reason this method should be applicable both for isolation and detection of vaccine antigen—hemagglutinin as well as influenza virus—because hemagglutinin is found on the surface of the influenza virion [37]. Some other authors also described the importance of methods for quantification of vaccine hemagglutinin. *Legastelois et al.* described ELISA assays, where HA detection was 1.9 μg/mL, as opposed to the 5 μg/mL quantitation limit generally accepted for the standard single-radial-immunodiffusion (SRID) assay, the approved technique for quantifying HA content in influenza vaccines [38]. *Bousse* *et al.* described a method which was applied to quantitate type A influenza viruses in solution, and focused on two early steps of vaccine preparation, firstly, detection and quantitation of virus harvested from eggs and cell cultures and, secondly, virus quantitation following inactivation [39].

## 3. Experimental

### 3.1. Chemicals

Tris(2-carboxyethyl)phosphine (TCEP) was supplied by Molecular Probes (Eugene, OR, USA). Co(NH_3_)_6_Cl_3_ and other chemicals were supplied by Sigma Aldrich (St. Louis, MO, USA) unless indicated otherwise. Deionised water passed demineralization by reverse osmosis using Aqua Osmotic 02 (Aqua Osmotic, Tisnov, Czech Republic) and further it was purified using Millipore RG (MiliQ water, 18 MΏ, Millipore Corp., Billerica, MA USA). Deionized water was used to rinse, wash and prepare buffers. Stock solutions were prepared with water of ACS purity.

### 3.2. Hemagglutinin

The vaccine Vaxigrip^®^ (Sanofi Pasteur, Lyon, France) was used as the sample of influenza hemagglutinin. Vaxigrip^®^ is inactivated trivalent influenza vaccine. Sterile suspension contain three influenza strains (A/California/7/2009 (H1N1), A/Victoria/361/2011 (H3N2) and B/Wisconsin/1/2010), cultivated on fertilised eggs, concentrated, purified by zone centrifugation in a sucrose gradient, split, inactivated and diluted in phosphate buffered saline solution. Vaxigrip^®^ contains 15 micrograms of all three HAs per 0.5 mL (90 µg/mL HA).

### 3.3. Preparation of QDs (CdTe)

CdTe QDs were prepared according to a slight modification of the method published by Duan *et al.* [40]. Briefly, cadmium acetate dihydrate (Cd(OAc)_2_·2H_2_O, 0.0267 g) was dissolved in ACS water (44 mL) and trisodium citrate dihydrate (100 mg) was added with stirring. A solution of Na_2_TeO_3_ (0.0055 g) in water (1.25 mL) was poured into the first solution, followed by 3-mercaptopropionic acid (100 µL, 1.14 mM). Afterwards, solid NaBH_4_ (50 mg) was added with vigorous stirring and hydrogen evolution was observed, followed by a color change of the solution to slightly yellow. After 30 min of stirring, an aliquot of solution (2 mL) was heated in a glass vial in a Multiwave 3000 Microwave Reaction System (Anton Paar, Graz, Austria) using a 64MG5 rotor. Reaction conditions were as follows: power 300 W, temperature 120 °C and time 18 min. Obtained CdTe QDs were stored in the dark at 4 °C.

### 3.4. Labelling of Vaxigrip^®^ HA by CdTe QDs

Vaxigrip^®^ (500 µL containing 45 µg of HA) was reduced and washed with water (5 × 400 µL) on an Amicon 3k centrifugal filter device (Millipore) and mixed with a solution of prepared QDs (500 µL). This mixture was shaken for 24 h at room temperature (Biosan Orbital Shaker OS-10, Biosan Ltd. Riga, Latvia). The volume of solution was reduced to 100 µL on an Amicon Ultra 3k centrifugal filter, diluted with water and washed five times using a 5417R centrifuge (Eppendorf, Hamburg, Germany). The washed sample was diluted to 1 mL with ACS water and used for measurements. 

### 3.5. Characterization of vaxi HA and HA-CdTe Complex by Gel Electrophoresis

Sodium dodecyl sulphate polyacrylamide gel electrophoresis (SDS-PAGE) was performed using a Mini Protean Tetra apparatus with gel dimensions of 8.3 × 7.3 cm (Bio-Rad, Hercules, CA, USA). First 12.5% (m/V) running, then 5% (m/V) stacking gel was poured. The gel was prepared from 30% (m/V) acrylamide stock solution with 1% (m/V) bisacrylamide. The polymerization of the running or stacking gels was performed at room temperature for 30 min. Prior to analysis the samples were mixed with non-reduction sample buffer in a 2:1 ratio. The samples were incubated at 93 °C for 3 min, and the sample was loaded onto a gel. For determination of the molecular mass, the “Precision plus Protein Standards” protein ladder from Bio-Rad was used. The electrophoresis was run at 150 V for 1 h at laboratory temperature (23 °C) (Power Basic, Bio-Rad) in tris-glycine buffer (0.025 M trizma-base, 0.19 M glycine and 3.5 mM SDS, pH = 8.3). Then the gels were stained Coomassie-blue. The rapid Coomassie-blue staining procedure was adopted from Wong *et al.* [41].

### 3.6. Isolation of HA-QDs by Glycan Modified MPs Using a Robotic Pipetting Station

#### 3.6.1. Robotic Pipetting Station

An ep*Motion* 5075 (Eppendorf, Hamburg, Germany) automated pipetting station, was used for CdTe labeled HA isolation prior to electrochemical analysis. The isolation process was computer controlled. The program sequence was edited and the station was supervised by pEditor 4.0. Transfer of species was provided by a robotic arm with pipetting adaptors (TS50, TS300 – numeric labeling refers to the maximal pipetting volume in µL) and a gripper for microplate transport (TG-T), was placed at the A1 position. Tips were placed in positions A3 (300 µL tips) and A2 (50 µL tips). At position A4 a Thermo mixer was located. At position B1, a module reservoir for washing solutions and waste were placed. Position C1 was thermostated (PCR 96 thermoadapter). A thermorack for 24 × 1.5–2 mL microtubes (Position B3) was used to store working solutions (buffers). For sample preparation 96-well PCR plates (microplates) with a maximal well volume of 200 μL were used. The principal position of the microplate was C1. During washing and at the end of isolation, the MPs were forced to the well bottoms using a magnetic adapter (Promega, Madison, WI, USA) (position C4). 

#### 3.6.2. The Automated Isolation Procedure

The first step of the automatic isolation involved pipetting of Dynabeads^®^ M-270 Streptavidin (10 μL), supplied by Life Technologies (Carlsbad, CA, USA), into microplate wells (PCR 96, Eppendorf). Then the plate was subsequently transferred to a magnet. Stored solution was aspirated out of the MPs and the MPs were washed three times with phosphate buffer (PB, 0.3 M, pH 7.4, made from NaH_2_PO_4_ and Na2HPO4, 100 μL). After that biotinyled multivalent-glycan (01-039a [Neu5Acα2-6Galβ1-4GlcNAcβ1-PAA-biotin], 20 μL) which was used according to *Suenaga at al.* [25], supplied by GlycoTech (Gaithersburg, MD, USA), were added to each of the wells and incubated (30 min, 25 °C, 400 rpm). In the next step each well was washed three times with PB (100 μL and sample (vaccine HA labeled by CdTe, 20 μL) was added. The mixture in each well was further incubated (400 rpm, optimized conditions) and washed three-times with PB (100 μL). In the last step PB (35 μL) was added followed by ultrasound needle treatment (2 min). The plate was transferred to the magnet, where the supernatant was pipetted out of MPs into the new wells and electrochemically analyzed. 

### 3.7. Electrochemical Detection of CdTe and HA-CdTe Complex

Cd (CdTe) itself and Cd from HA-CdTe complex was detected by differential pulse anodic stripping voltammetry (DPASV). Measurements were carried on a 663 VA Stand (Metrohm, Herisau, Switzerland) with an electrochemical cell with a three electrode set-up. A hanging mercury drop electrode (HMDE) with a drop area of 0.4 mm^2^ was used as the working electrode, an Ag/AgCl/3M KCl as a reference electrode and a platinum electrode was the auxiliary electrode. Samples were deoxygenated prior to each measurement by purging with argon (99.999%). Acetate buffer (0.2 M CH_3_COONa + CH_3_COOH, pH 5.0) was used as a background electrolyte and was replaced after each measurement. The parameters of the measurement were as it follows: purging time 100 s; deposition potential −0.85 V; equilibration time 2 s; modulation time 0.057 s; interval time 0.2 s; initial potential −0.85 V; end potential −0.4 V; step potential 0.005 V; modulation amplitude 0.0250 V; volume of measurement cell: 1 mL (5 μL sample; 995 µL acetate buffer). GPES 4.9 software was used for data processing.

### 3.8. Electrochemical Detection of vaxi HA and HA-CdTe Complex

HA itself and HA from HA-CdTe complex were detected by the adsorptive transfer technique connected with a differential pulse voltammetric method (AdT DPV) Brdicka reaction [42]. All measurements were performed with a 663 VA Stand instrument (Metrohm) with a cooled electrochemical cell, equipped with a three electrode set-up. The temperature of the measurement cell was controlled at 4 °C by a Julabo F25 instrument (JulaboDE, Seelbach, Germany). The three-electrode setup was composed of a hanging mercury drop electrode (HMDE) with a drop area of 0.4 mm^2^ as the working electrode, an Ag/AgCl/3M KCl reference electrode and a platinum electrode as the auxiliary one. Prior to measurements the analysed samples were deoxygenated by purging with argon (99.999%) for 120 s. The Brdicka supporting electrolyte contained 1 mM Co(NH_3_)_6_Cl_3_ and 1 M ammonia buffer (NH_3_(aq) and NH_4_Cl, pH = 9.6) and was changed after each analysis. The parameters of the measurement were as it follows: initial potential −0.7 V; end potential −1.75 V; modulation time 0.057 s; time interval 0.2 s; step potential 0.002 V; modulation amplitude 0.025 V; sample volume 5 µL; buffer volume 1500 µL. Software GPES 4.9 was used for data analysis.

### 3.9. Descriptive Statistics

Data were processed using MICROSOFT EXCELs (Microsoft Corp., Redmond, WA, USA) and STATISTICA.CZ Version 8.0 (StatSoft, Prague, Czech Republic). The results are expressed as mean ± SD unless noted otherwise. The detection limits (3 signal/noise, S/N) were calculated according to Long and Winefordner [43], where N was expressed as a standard deviation of noise determined in the signal domain, unless stated otherwise.

## 4. Conclusions

In this study we designed and optimized method for automated isolation and detection of vaccine influenza hemagglutinin labeled with CdTe quantum dots. For the detection of HA-CdTe complex two voltammetric methods were used. The effect of various conditions (glycan concentration, time and temperature of glycan and HA-CdTe mixing on the detected HA and Cd signals were investigated. These results demonstrate that the automatic isolation procedure coupled with the usage of quantum dots as an electrochemical label is suitable for vaccine influenza detection. Our platform may be applied for analysis of other pathogens, based on specific receptor binding and magnetic isolation processes.
